# Oncogenes in Cancer: Using the Problem as Part of the Solution

**DOI:** 10.3390/cancers12113373

**Published:** 2020-11-14

**Authors:** Ignacio Gil-Bazo

**Affiliations:** 1 Department of Oncology, Clínica Universidad de Navarra, 31008 Pamplona, Spain; igbazo@unav.es; Tel.: +34-948-255400; Fax: +34-948-255500; 2 Program of Solid Tumors, Center for Applied Medical Research, University of Navarra, 31008 Pamplona, Spain; 3 IdiSNA, Navarra Institute for Health Research, 31008 Pamplona, Spain; 4 Centro de Investigación Biomédica en Red de Cáncer (CIBERONC), 28029 Madrid, Spain

Human cancer is considered to have a multifactorial origin. The exposure to certain environmental, occupational or social carcinogens such as ultraviolet irradiation [1], asbestos [2,3], radon [3] or tobacco [2], among others, is well documented to increase the individual risk of developing a number of neoplasms. In addition, a growing concern is infection by specific viruses (EBV [4], VIH [5], HPV [6], HCV [7]…) as other sources of cancer-related factors.

However, human malignancies have also been considered a genomic condition [8]. In fact, genome instability and mutations are one of the well-recognized hallmarks of cancer [9]. More specifically, a large number of hereditary cancer syndromes can cause a higher individual predisposition to develop cancer [10]. Most of these syndromes are generated by germline mutations in cancer suppressor genes, and they may cause the appearance of different tumor types. Overall, it is estimated that up to 10% of all new cancer cases are attributed to inherited genetic alterations [10].

In contrast, many other malignancies develop in individuals with unknown inherited genomic factors, increasing their susceptibility to cancer, but in whom a somatic mutation in an oncogene acts as a driver genomic alteration facilitating tumor development [11]. Identifying these cancer-driver mutations has become key to providing tailored treatment in a precision-medicine fashion [12].

Over the last 20 years, several cancer-driver mutations have been identified and, more importantly, targeted agents have become a new standard of care for treating such patients [13]. Some of these somatic mutations are generated due to exposure to different toxics such as tobacco [14]. More importantly, it has been demonstrated that in different tumor types, when a driver mutation is identified and can be targeted, there is a significant increase in overall survival as well as a remarkable improvement in the quality of life of these patients [15].

Interestingly enough, in some cancer subtypes, the presence of certain driver mutations may not only predict the benefits associated with tailored treatment but may also preclude the activity of another recently incorporated cancer treatment strategy, such as immune checkpoint inhibitors [16], stressing the need for a proper identification of those activating mutations.

More recently, next-generation sequencing techniques have facilitated a broader identification of potential therapeutic targets. Several basket clinical trials have shown the benefit of particular targeted compounds against solid tumors of different origins as long as have a specific pathogenic molecular alteration. This fact has definitely contributed to a profound change in the regulatory procedures leading to the approval, for the first time, of tumor-agnostic drugs based on particular molecular features [17].

In this Special Issue, world-wide experts in the field of cancer genomics are invited to contribute original articles or comprehensive reviews reporting and discussing the more recent advances in the role of oncogenes in cancer development and progression. Moreover, research describing the molecular activation of different biological pathways in oncogene-addicted neoplasms results of particular interest for this Special Issue. In addition, manuscripts updating and discussing the use of more sensitive genomic diagnostic tools in tissue samples as well as liquid biopsy assays and the implementation of new therapeutic strategies in the field of precision medicine for cancer patients are welcome.
